# Nelfinavir Synergizes with Cisplatin and Promotes GSDME-Associated Lytic Cell Death in Platinum-Resistant Ovarian Cancer Cells

**DOI:** 10.3390/ph19101665

**Published:** 2026-10-21

**Authors:** Benjamin N. Forgie, Rewati Prakash, Desiree Marno, Farah H. Abdalbari, Edith Zorychta, Abu Shadat M. Noman, Alicia A. Goyeneche, Lucy Gilbert, Julia V. Burnier, Carlos M. Telleria

**Affiliations:** 1 Department of Pathology, McGill University, Montreal, QC H3A 2B4, Canada; benjamin.forgie@mail.mcgill.ca (B.N.F.); farah.abdalbari@mail.mcgill.ca (F.H.A.);; 2 Cancer Research Program, Research Institute of the McGill University Health Centre, Montreal, QC H4A 3J1, Canada; alicia.goyeneche@affiliate.mcgill.ca; 3 Department of Pharmacology and Therapeutics, McGill University, Montreal, QC H3G 1Y6, Canada; 4 Department of Biochemistry and Molecular Biology, Chittagong University, Chittagong 4331, Bangladesh; 5 Division of Gynecologic Oncology, Research Institute of the McGill University Health Centre, Montreal, QC H3H 2R9, Canada; 6 Gerald Bronfman Department of Oncology, McGill University, Montreal, QC H4A 3T2, Canada

**Keywords:** ovarian cancer, cisplatin, nelfinavir, drug repurposing, combination therapy, platinum resistance, lytic cell death, gasdermin E, endoplasmic reticulum stress

## Abstract

**Background/Objectives:** Platinum resistance limits the efficacy of ovarian cancer treatment and arises through multiple mechanisms, including enhanced DNA repair, activation of stress responses, and increased survival signaling. Nelfinavir (NFV), an HIV protease inhibitor with reported anti-tumor activity, is a candidate for drug repurposing, but its interaction with cisplatin (CDDP) in ovarian cancer has not been established. Here, we investigated whether NFV enhances CDDP cytotoxicity in platinum (Pt)-resistant ovarian cancer cells and characterized the associated cell death response. **Methods:** Drug interaction was assessed using cell vitality and viability assays and Loewe additivity modeling together with Bliss and ZIP analyses. Cell death was characterized by Annexin V/7-AAD staining, mitochondrial membrane potential analysis, lactate dehydrogenase (LDH) release, immunoblotting, pharmacological caspase inhibition, and bulk mRNA sequencing with differential expression and gene set enrichment analyses. **Results:** NFV synergistically enhanced CDDP cytotoxicity across three Pt-resistant ovarian cancer cell lines, reducing CDDP IC50 values by 4.2–5.8-fold and producing mean Loewe synergy scores of approximately 25–30. Combination treatment increased LDH release and was accompanied by caspase-8, caspase-3, PARP, and the pore-forming death effector gasdermin E (GSDME) cleavage. Caspase inhibition attenuated viability loss and GSDME processing. Transcriptomic analysis identified enrichment of stress- and death-associated programs, including the unfolded protein response (UPR), apoptosis, reactive oxygen species (ROS)-associated signaling, and TNF/NF-κB signaling. **Conclusions:** Together, these findings demonstrate a reproducible synergistic interaction between CDDP and NFV in Pt-resistant ovarian cancer cells and characterize an associated lytic cell death phenotype involving apoptotic signaling and accompanied by GSDME processing. These findings support further investigation of NFV as a repurposed combination partner to enhance CDDP cytotoxicity in Pt-resistant ovarian cancer.

## 1. Introduction

Ovarian cancer is the most lethal gynecological malignancy and remains a major clinical challenge due to frequent diagnosis at an advanced stage and high rates of disease recurrence [1,2]. Standard-of-care treatment comprises cytoreductive surgery in combination with platinum (Pt)-based chemotherapy [3]. Despite initial responsiveness to Pt-based chemotherapy, most patients with ovarian cancer ultimately develop recurrent Pt-resistant disease, underscoring the need for strategies that enhance treatment efficacy [4]. Pt resistance arises through a network of adaptive changes that collectively reduce treatment sensitivity and limit cell death; these include mechanisms such as dysregulation of Pt influx and efflux transporters [5], increased DNA damage repair capacity [6], activation of stress-adaptive programs [7,8], and engagement of pro-survival signaling pathways such as PI3K-Akt while simultaneously weakening pro-death signaling [4,9,10]. As a result, Pt-resistant cells may tolerate substantial Pt-induced damage yet fail to convert that stress into efficient cell death [11]. Accordingly, there is considerable interest in identifying agents that can enhance the cytotoxic effects of Pt drugs and interfere with or overcome these adaptive survival responses in ovarian cancer cells.

Nelfinavir (NFV), a human immunodeficiency virus (HIV) protease inhibitor [12], has emerged as a drug-repurposing candidate in oncology [13] due to its reported anti-tumor activity across multiple cancer types [14]. We have previously demonstrated that NFV exerts cytotoxic effects against high-grade serous ovarian cancer (HGSOC) cells through engagement of pathways linked to the unfolded protein response (UPR), DNA damage, and pro-apoptotic signaling [15]. Additionally, NFV has been shown to alter Akt- and Erk-mediated pro-survival signaling [15]. Together, these findings provide a rationale for investigating whether NFV can enhance the cytotoxicity of Pt-based drugs in ovarian cancer cells. However, whether NFV interacts with cisplatin (CDDP), the first-generation Pt agent used worldwide [16,17], to kill Pt-resistant ovarian cancer cells remains unknown.

Pt drugs such as CDDP primarily exert their anti-cancer effects through the formation of detrimental intra- and inter-strand DNA crosslinks [9,18], which in turn activate the DNA damage response [19] and culminate in engagement of the intrinsic apoptotic pathway and activation of executioner caspases-3 and -7 [9,18]. In some contexts, apoptotic signaling may also be reinforced through activation of initiator caspases such as caspase-8 [20], which can link upstream cell death signaling to mitochondrial apoptotic amplification through cleavage of Bid, a pro-apoptotic BH3-only protein member of the BCL-2 family of genes [21], and through direct executioner caspase cleavage [20,22]. While caspase-3 is canonically associated with apoptotic execution, it can also function as a molecular branch point linking apoptosis to lytic cell death [23,24]. Gasdermins (GSDMs) are a family of pore-forming proteins best known as executioners of pyroptosis, a regulated lytic cell death program characterized by the formation of gasdermin (GSDM) protein pores in the plasma membrane. These small pores range from 10–20 nm in size and permit ion flux and leakage of small intracellular molecules [25,26]. Following release of these intracellular materials, terminal plasma membrane rupture is mediated by ninjurin-1 (NINJ1) [27], which oligomerizes in the plasma membrane to form large ring-like openings, ranging from 10–100 nm, that enable the leakage of larger molecules such as lactate dehydrogenase (LDH), a marker of lytic cell death [28]. Among the GSDM family, gasdermin E (GSDME) can be cleaved by caspase-3, releasing its pore-forming N-terminal fragment. In cells expressing sufficient GSDME, this provides a mechanism through which apoptotic caspase activity can acquire lytic features, resulting in membrane permeabilization and eventual complete loss of plasma membrane integrity [23,24,29]. Accordingly, the outcome of caspase-3 activation may depend not only on the strength of apoptotic signaling, but also on cellular determinants such as GSDME expression and the upstream signaling environment in which caspase-3 is activated [29]. Combination therapies capable of amplifying caspase activity may therefore influence not only the extent of cell death, but also whether apoptotic signaling progresses toward a more lytic terminal phenotype.

In this study, we evaluated NFV as a potential repurposed combination partner for CDDP in Pt-resistant ovarian cancer cells. We report a synergistic cytotoxic interaction between CDDP and NFV and characterize the associated lytic cell death response, which occurs in a cellular context marked by apoptotic signaling, GSDME processing, endoplasmic reticulum (ER) stress, and DNA damage-associated changes. Collectively, these findings help characterize the cellular response of Pt-resistant ovarian cancer cells to combined CDDP/NFV treatment and identify mechanistic relationships that warrant further validation.

## 2. Results

### 2.1. NFV and CDDP Synergistically Reduce Viability in Pt-Resistant Ovarian Cancer Cells

**Figure 1:**
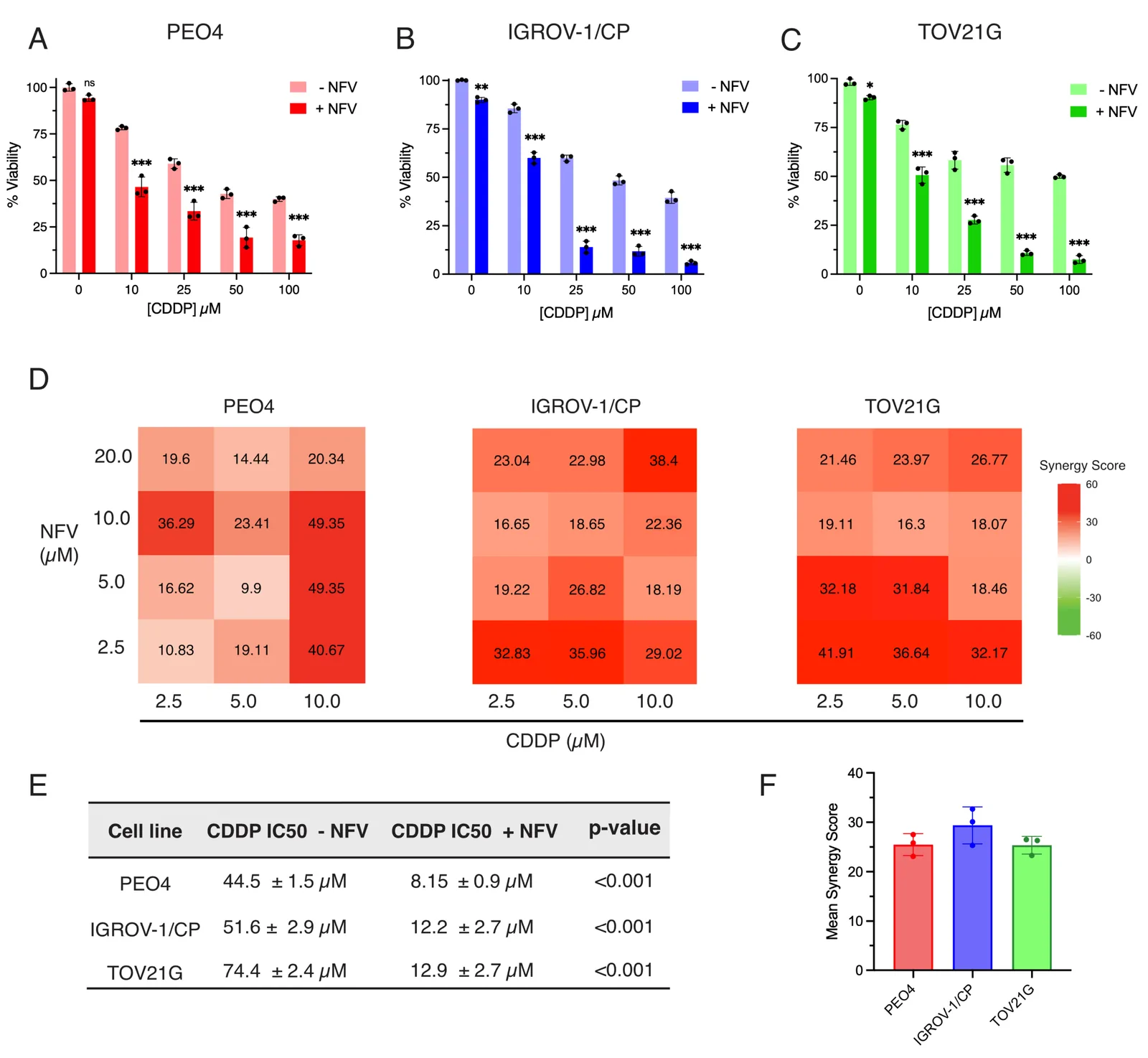
Dose–response and synergy analysis of combined cisplatin and nelfinavir treatment in Pt-resistant ovarian cancer cells. (**A**–**C**) Dose–response graphs showing cell viability following treatment with increasing concentrations of cisplatin (CDDP) alone or in combination with 20 µM nelfinavir (NFV) in PEO4, IGROV-1/CP, and TOV21G cells, respectively. NFV alone is included at 0 µM CDDP. (**D**) Loewe synergy heatmaps generated using SynergyFinder analysis software version 3.20.0, showing synergy scores for all tested CDDP/NFV dose combinations in PEO4, IGROV-1/CP and TOV21G cells. Scores less than −10 are considered to be antagonistic, scores from −10 to 10 are considered additive, and scores greater than 10 are considered synergistic. (**E**) Mean IC50 values for CDDP alone and CDDP when in combination with 20 µM NFV in each cell line. (**F**) Mean Loewe synergy scores calculated by averaging the scores from all individual dose combinations in each cell line. Data are presented as mean ± SD from three independent experiments. Statistical significance in panels A-C was determined by two-way ANOVA followed by Šídák’s multiple comparisons test, comparing cells treated with and without NFV at each CDDP concentration. ns, not significant (*p* ≥ 0.05); * *p* < 0.05, ** *p* < 0.01, *** *p* < 0.001.

To determine whether NFV enhances the cytotoxic effects of CDDP in ovarian cancer cells, PEO4, IGROV-1/CP, and TOV21G cells were exposed to increasing concentrations of CDDP (0, 10, 25, 50, or 100 µM) for 3 h, followed by culture in drug-free medium or medium containing NFV (20 µM) for 72 h. At 20 µM, NFV alone produced a modest reduction in viability across all three cell lines (Figure 1A–C). In contrast, combining NFV with CDDP consistently enhanced CDDP cytotoxicity, resulting in lower viability across the tested CDDP concentrations than with CDDP alone (Figure 1A–C). To quantify NFV’s effect on CDDP toxicity, we compared CDDP IC50 values, defined as the concentration of CDDP required to reduce cell viability by 50%, in the absence or presence of 20 µM NFV. The addition of NFV reduced the CDDP IC50 by approximately 5.5-fold in PEO4 cells, 4.2-fold in IGROV-1/CP cells, and 5.8-fold in TOV21G cells (Figure 1E). Synergy analysis using the Loewe additivity model [30] further supported a cooperative interaction between the two agents. Loewe scores were positive across the tested concentration combinations in all three cell lines (Figure 1D), with mean Loewe synergy scores of approximately 25–30, consistent with synergy in each model tested (Figure 1F). Additional analyses using Bliss independence and ZIP reference models further supported overall synergy in all three cell lines, with synergy scores for individual dose combinations ranging from additive to strongly synergistic (Supplementary Figure S1). Together, these findings demonstrate a synergistic cytotoxic interaction between NFV and CDDP across the three Pt-resistant ovarian cancer cell lines studied.

### 2.2. Combined CDDP/NFV Treatment Induces Lytic Cell Death Accompanied by Apoptotic Signaling and GSDME Processing

**Figure 2:**
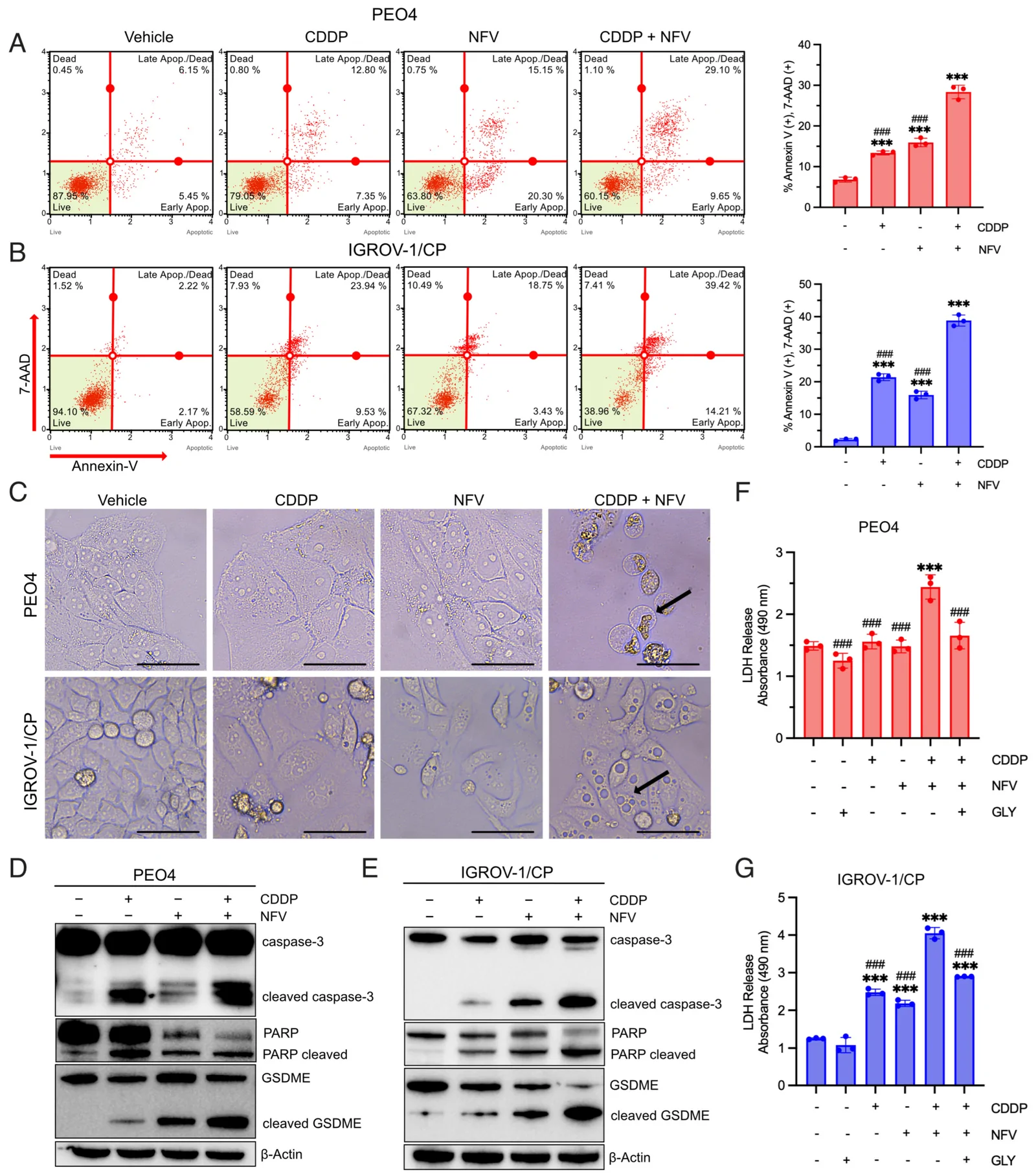
Cell death characteristics and GSDME processing following combined cisplatin and nelfinavir treatment. PEO4 and IGROV-1/CP cells were treated with vehicle for 72 h, cisplatin (CDDP; 10 µM) for 3 h followed by drug-free medium for 72 h, nelfinavir (NFV; 20 µM) for 72 h, or CDDP for 3 h followed by NFV for 72 h, as indicated. (**A**,**B**) Representative Annexin V/7-AAD microcapillary-flow cytometry plots and quantification of Annexin V-positive/7-AAD-positive cells in PEO4 (**A**) and IGROV-1/CP (**B**) cells. (**C**) Representative brightfield images of PEO4 and IGROV-1/CP cells following treatments. Black arrows indicate enlarged, ballooned cells in PEO4 and swollen/vacuolated cells in IGROV-1/CP following combination treatment. Scale bars = 50 µm. (**D**,**E**) Immunoblot analysis of caspase-3, PARP, and gasdermin E (GSDME) cleavage in PEO4 (**D**) and IGROV-1/CP (**E**) cells following treatment. (**F**,**G**) Lactate dehydrogenase (LDH) release assay in PEO4 (**F**) and IGROV-1/CP (**G**) cells following CDDP/NFV treatment in the absence or presence of 5 mM glycine (GLY). Data are presented as mean ± SD from three independent experiments. Statistical significance was determined by one-way ANOVA with Tukey’s post hoc test. *** *p* < 0.001 versus vehicle; ### *p* < 0.001 versus CDDP plus NFV.

Having established synergy across three Pt-resistant ovarian cancer cell lines, we selected PEO4 and IGROV-1/CP cells for further mechanistic characterization. These cell lines represent distinct models of acquired Pt resistance, with PEO4 derived from a patient with Pt-resistant disease [31], and IGROV-1/CP generated through chronic cisplatin exposure in vitro [32]. To explore the cell death response to the combination of CDDP with NFV, we assessed Annexin V/7-AAD staining, cellular morphology, LDH release, and cleavage of key cell death effectors following treatment. In both PEO4 and IGROV-1/CP cells, microcapillary-flow cytometric analysis showed that the combination treatment increased the proportion of Annexin V- and 7-AAD-positive cells relative to vehicle and single-agent controls, consistent with enhanced cell death accompanied by loss of membrane integrity (Figure 2A,B). Representative brightfield images supported a mixed death phenotype following combination treatment; in addition to detached, shrunken cells consistent with apoptosis, PEO4 cells displayed enlarged detached cells with ballooning morphology, whereas IGROV-1/CP cells exhibited prominent vacuolization and cellular swelling, morphological features reported in pyroptotic/lytic cell death [33,34] (Figure 2C). To further define the molecular features of this response, we examined cleavage of caspase-3 and its downstream target PARP [35], and of GSDME. In both cell lines, immunoblots showed increased caspase-3, PARP, and GSDME cleavage following combination treatment, consistent with apoptotic signaling accompanied by GSDME processing (Figure 2D,E; Supplementary Figure S2A). In parallel, the drug combination significantly increased LDH release relative to either monotherapy in both cell lines, further supporting loss of plasma membrane integrity (Figure 2F,G). To determine whether terminal membrane rupture contributed to the observed lytic phenotype, we co-treated CDDP/NFV-treated cells with glycine, a widely used cytoprotective agent that limits plasma membrane rupture during lytic cell death, likely by inhibiting the NINJ1 membrane clustering [36,37]. Glycine significantly reduced LDH release following combination treatment, supporting a contribution of terminal membrane disruption to the observed death phenotype (Figure 2F,G). Together, these findings indicate that CDDP/NFV treatment induces a lytic death phenotype accompanied by apoptotic signaling and GSDME cleavage.

### 2.3. Combined CDDP/NFV Treatment Is Associated with Caspase-8 Cleavage and Mitochondrial Apoptotic Signaling

**Figure 3:**
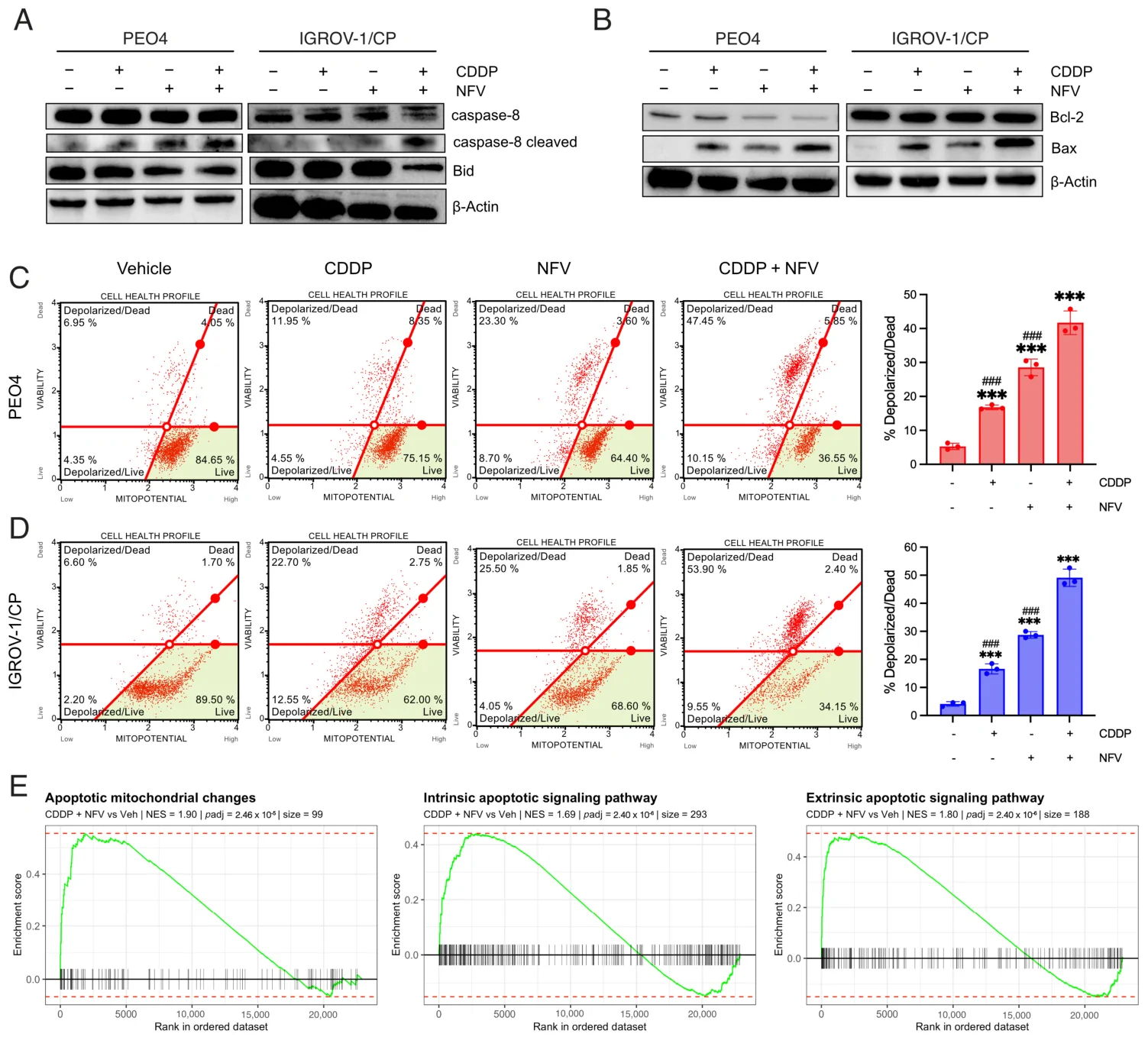
Caspase-8 and mitochondrial apoptotic signaling following combined cisplatin and nelfinavir treatment. PEO4 and IGROV-1/CP cells were treated with vehicle (72 h), CDDP (10 µM, 3 h) followed by drug-free medium (72 h), NFV (20 µM, 72 h), or CDDP (10 µM, 3 h) followed by NFV (20 µM, 72 h), as indicated. (**A**) Immunoblot analysis of caspase-8 and Bid in PEO4 and IGROV-1/CP cells following treatment. (**B**) Immunoblot analysis of Bcl-2 and Bax in PEO4 and IGROV-1/CP cells following treatment. β-Actin was used as a loading control. (**C**,**D**) Representative mitochondrial membrane potential microcapillary-flow cytometry plots and quantification of depolarized/dead cells in PEO4 (**C**) and IGROV-1/CP (**D**) cells following treatment. (**E**) Gene set enrichment plots showing enrichment of apoptotic mitochondrial changes, intrinsic apoptotic signaling, and extrinsic apoptotic signaling in CDDP/NFV-treated IGROV-1/CP cells relative to vehicle controls after 24 h. Red dashed lines indicate the maximum and minimum running enrichment scores, while the solid black horizontal line indicates an enrichment score of zero, and black vertical ticks represent the positions of genes belonging to each gene set within the ranked gene list. Data in panels C and D are presented as mean ± SD. Statistical significance was determined by one-way ANOVA with Tukey’s post hoc test. *** *p* < 0.001 versus vehicle; ### *p* < 0.001 versus CDDP/NFV. Gene set enrichment analysis in panel E was performed using fgsea, as described in Section 4.10.

Because combined CDDP/NFV treatment was accompanied by apoptotic signaling and a lytic cell death phenotype, we examined caspase-8 and mitochondrial changes. Caspase-8 can couple upstream death signaling to the mitochondrial apoptotic pathway through Bid processing into cleaved Bid, a pro-apoptotic *BCL2* family member [21]. Consistent with this, the CDDP/NFV treatment increased caspase-8 cleavage in both PEO4 and IGROV-1/CP cells relative to vehicle and single-agent controls (Figure 3A). In parallel, we observed a reduction in full-length Bid, consistent with Bid processing (Figure 3A). To evaluate mitochondrial integrity in drug-treated cells, we examined the relative levels of the pro-apoptotic protein Bax and the anti-apoptotic protein Bcl-2, as the Bax/Bcl-2 ratio is a major determinant of outer mitochondrial membrane (OMM) permeabilization [38]. Combined CDDP and NFV increased the Bax/Bcl-2 ratio in both cell lines, indicating a shift toward a pro-apoptotic mitochondrial state (Figure 3B) [38]. Consistent with this observation, analysis of IMM potential revealed an increased proportion of cells with a depolarized IMM following combination treatment (Figure 3C,D). Transcriptomic analysis further supported activation of these apoptotic programs. Gene ontology biological process enrichment [39,40] demonstrated significant positive enrichment of apoptotic mitochondrial changes, intrinsic apoptotic signaling, and extrinsic apoptotic signaling in IGROV-1/CP cells treated with both CDDP and NFV relative to vehicle-treated controls (Figure 3E). Together, these findings are consistent with caspase-8-associated and mitochondrial apoptotic signaling following combination of CDDP and NFV.

### 2.4. Pharmacological Caspase Inhibition Attenuates CDDP/NFV-Associated Loss of Viability and GSDME Processing

**Figure 4:**
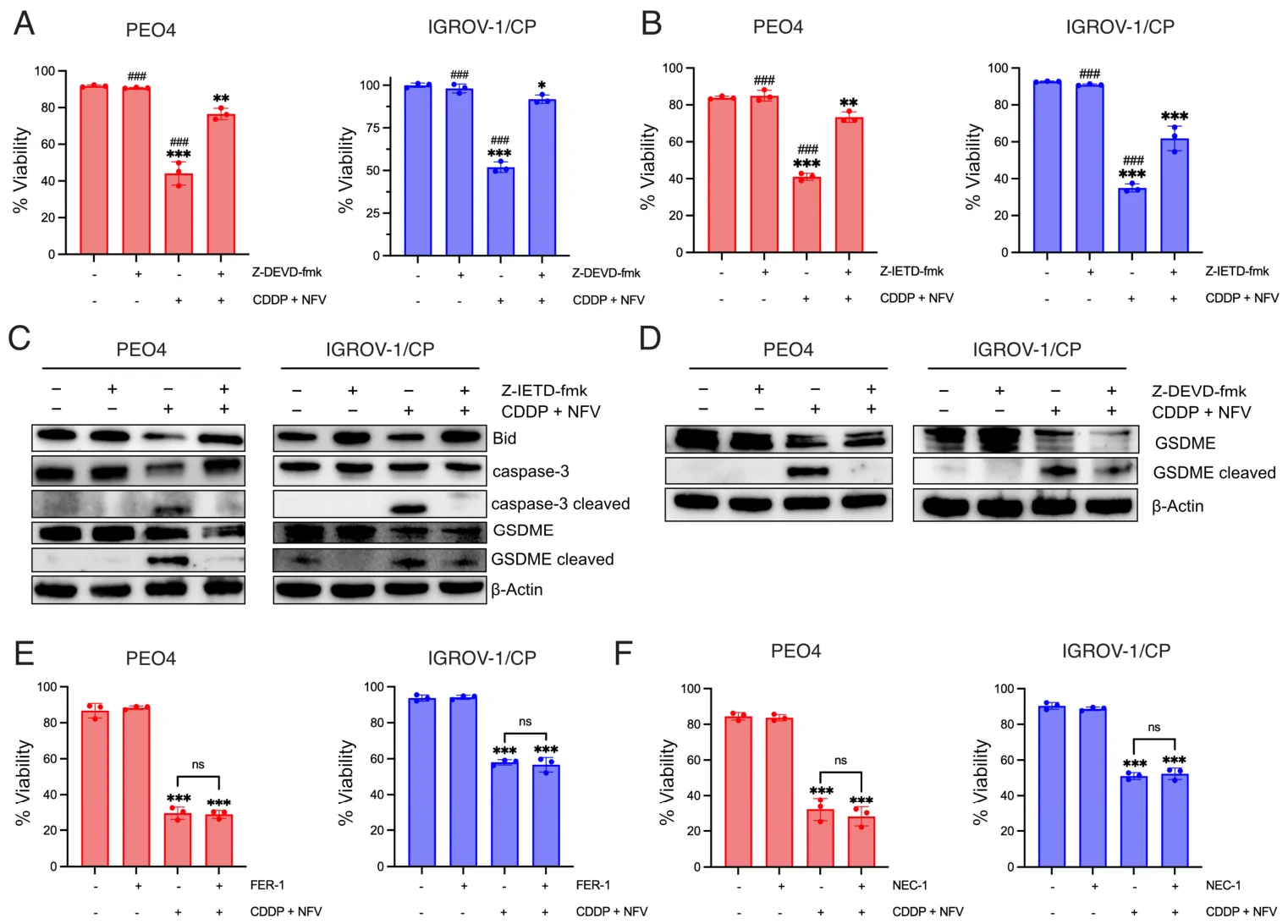
Effects of caspase inhibition on cisplatin/nelfinavir-associated viability loss and GSDME processing. PEO4 and IGROV-1/CP cells were treated with vehicle or CDDP (10 µM, 3 h) followed by NFV (20 µM, 72 h), in the absence or presence of Z-DEVD-fmk (caspase-3 inhibitor; 100 µM), Z-IETD-fmk (caspase-8 inhibitor; 100 µM), ferrostatin-1 (FER-1, ferroptosis inhibitor; 1 µM), or necrostatin-1 (NEC-1, necroptosis inhibitor; 10 µM), as indicated. (**A**,**B**) Cell viability in PEO4 and IGROV-1/CP cells following treatment with CDDP + NFV in the absence or presence of Z-DEVD-fmk (**A**) or Z-IETD-fmk (**B**). (**C**) Immunoblot analysis of Bid, caspase-3, and gasdermin E (GSDME) in PEO4 and IGROV-1/CP cells treated with CDDP + NFV in the absence or presence of Z-IETD-fmk. (**D**) Immunoblot analysis of GSDME cleavage in PEO4 and IGROV-1/CP cells treated with CDDP/NFV in the absence or presence of Z-DEVD-fmk. (**E**,**F**) Cell viability in PEO4 and IGROV-1/CP cells following treatment with CDDP/NFV in the absence or presence of FER-1 (**E**) or NEC-1 (**F**). Data are presented as mean ± SD from three independent experiments. Statistical significance was determined by one-way ANOVA with Tukey’s post hoc test. * *p* < 0.05, ** *p* < 0.01, and *** *p* < 0.001 versus vehicle; ### *p* < 0.001 versus CDDP/NFV + Z-DEVD-fmk/Z-IETD-fmk; ns, not significant.

Having identified caspase-8, caspase-3, and GSDME cleavage following CDDP/NFV treatment, we next examined whether pharmacological inhibition of caspase-8 or caspase-3 altered the resulting cell death response. In both PEO4 and IGROV-1/CP cells, inhibition of caspase-3 with Z-DEVD-fmk significantly improved viability following the combination treatment, supporting a contribution of caspase-3 activity to the drug combination-induced death phenotype (Figure 4A). Similarly, treatment with the caspase-8 inhibitor Z-IETD-fmk improved viability in both cell lines, supporting a contribution of caspase-8 to this response (Figure 4B). Immunoblotting analysis supported this attenuation of viability loss, as inhibition of caspase-8 with Z-IETD-fmk reduced caspase-3 and GSDME cleavage and attenuated the reduction in full-length Bid (Figure 4C).

Inhibition of caspase-3 with Z-DEVD-fmk also reduced GSDME cleavage following CDDP/NFV treatment (Figure 4D; Supplementary Figure S2B). These findings provide pharmacological support for caspase involvement in the death response and GSDME processing. Because lytic cell death can result from regulated cell death pathways other than pyroptosis, we next asked whether ferroptosis and necroptosis contributed to the cell lysis observed following CDDP/NFV treatment. To test this, we treated cells with CDDP and NFV in the presence of ferrostatin-1, a radical-trapping antioxidant that inhibits lipid peroxidation to prevent ferroptosis [41], or necrostatin-1, an inhibitor of RIPK1-dependent necroptosis [42]. Neither inhibitor rescued cell viability in either cell line after CDDP/NFV treatment when used at concentrations reported to block ferroptosis and necroptosis in cancer cells [41,42] (Figure 4E,F). These negative pharmacological results did not provide evidence for a major contribution of ferroptosis or RIPK1-dependent necroptosis under these conditions; however, further investigation using genetic ablation approaches will be necessary to definitively exclude their involvement. Collectively, these findings provide pharmacological support for contributions of caspase-8 and caspase-3 to CDDP/NFV-induced lytic cell death and associate their activity with GSDME processing.

### 2.5. The Combination of CDDP and NFV Is Associated with Stress- and Death-Related Transcriptional Programs and Altered Akt- and DNA Damage-Associated Markers

**Figure 5:**
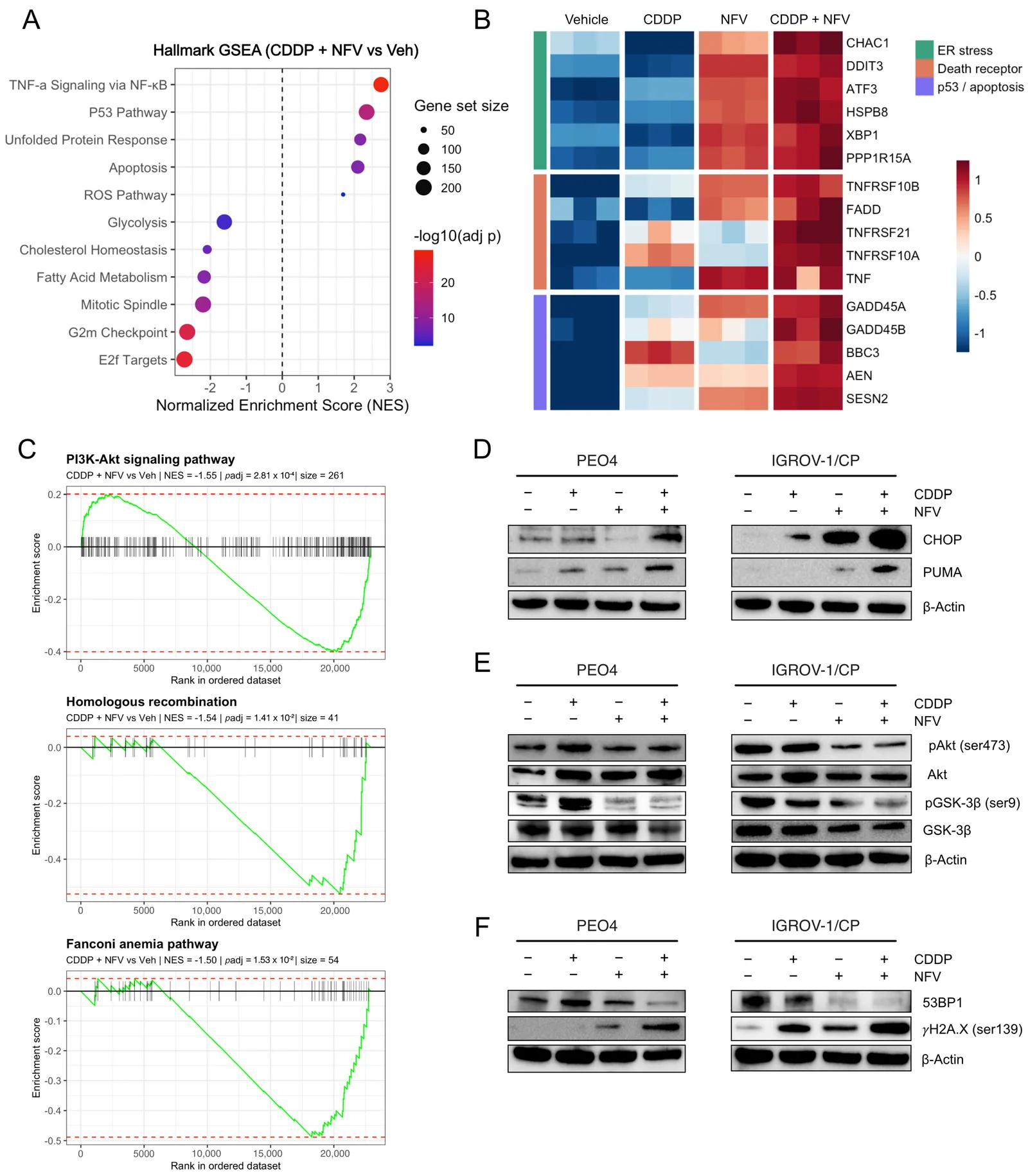
Transcriptomic and protein-level characterization of stress, survival, and DNA damage-associated responses to combined cisplatin and nelfinavir treatment. For RNA-seq panels (**A**–**C**), IGROV-1/CP cells were treated with vehicle (24 h), cisplatin (CDDP; 10 µM, 3 h) followed by drug-free medium (24 h), nelfinavir (NFV; 20 µM, 24 h), or CDDP (10 µM, 3 h) followed by NFV (20 µM, 24 h). For immunoblot panels (**D**–**F**), PEO4 and IGROV-1/CP cells were treated with vehicle (72 h), cisplatin (CDDP; 10 µM, 3 h) followed by drug-free medium (72 h), nelfinavir (NFV; 20 µM, 72 h), or CDDP (10 µM, 3 h) followed by NFV (20 µM, 72 h), as indicated. (**A**) Hallmark gene set enrichment analysis of CDDP/NFV-treated IGROV-1/CP cells relative to vehicle controls. Dot size represents gene set size, and color represents the -log10 adjusted *p*-value. (**B**) Heatmap showing Z-score-scaled expression of selected transcripts associated with ER stress, death receptor signaling, and p53/apoptosis across treatment groups in IGROV-1/CP cells. Columns represent individual biological replicates. Expression values are shown as row-scaled Z scores, with the color scale capped at ±1.25. (**C**) Gene set enrichment plots of selected KEGG pathways in CDDP plus NFV-treated IGROV-1/CP cells relative to vehicle controls. Normalized enrichment scores (NES), adjusted *p*-value, and gene set size are indicated. Red dashed lines indicate the maximum and minimum running enrichment scores, while the solid black horizontal line indicates an enrichment score of zero, and black vertical ticks represent the positions of genes belonging to each gene set within the ranked gene list. (**D**) Representative immunoblot analysis of CHOP and PUMA expression in PEO4 and IGROV-1/CP cells following treatment. (**E**) Representative immunoblot analysis of phosphorylated Akt (Ser473), total Akt, phosphorylated GSK-3β (Ser9), and total GSK-3β in PEO4 and IGROV-1/CP cells following treatment. (**F**) Representative immunoblot analysis of 53BP1 and γH2AX (Ser139) in PEO4 and IGROV-1/CP cells following treatment. β-actin was used as a loading control for the immunoblot analyses.

To place the apoptosis-focused transcriptomic analysis shown in Figure 3 in a broader transcriptional context, we next examined differential gene expression and Hallmark pathway enrichment in Pt-resistant ovarian cancer cells treated with CDDP/NFV relative to vehicle controls. Principal component analysis and sample-to-sample correlation analysis demonstrated appropriate replicate clustering and overall consistency across samples (Supplementary Figure S3). At the pathway level, Hallmark analysis revealed enrichment of stress- and death-associated pathways, including reactive oxygen species (ROS), apoptosis, the unfolded protein response (UPR), and TNF/NF-κB signaling (Figure 5A). Interestingly, the p53 signaling pathway was also enriched despite the mutant *TP53* background of IGROV-1/CP cells (Figure 5A). In contrast, pathways linked to glycolysis, cholesterol homeostasis, fatty acid metabolism, mitotic spindle, G2/M checkpoint, and E2F targets were negatively enriched (Figure 5A). Consistent with this broader stress-associated response, ROS production was observed following combination treatment in both PEO4 and IGROV-1/CP Pt-resistant ovarian cancer cells (Supplementary Figure S4). Overall, CDDP/NFV combination-treated Pt-resistant ovarian cancer cells exhibited a transcriptional profile enriched for stress- and death-associated programs and negatively enriched for several metabolic and proliferative programs. To examine these responses at the gene level, we generated an integrated heatmap of selected transcripts across vehicle-, CDDP-, NFV-, and CDDP/NFV-treated groups. Genes associated with endoplasmic reticulum (ER) stress, including *DDIT3* [43] (coding for CHOP), *ATF3* [44], and *XBP1* [45], were elevated following combination treatment (Figure 5B). Likewise, genes linked to DNA damage-associated pro-apoptotic signaling, including *GADD45A/B* [46], *BBC3* (coding for the pro-apoptotic protein PUMA) [47], and *AEN* [48], were also upregulated (Figure 5B). Notably, we also observed increased expression of transcripts related to extrinsic apoptotic and death receptor signaling, including *TNFRSF10A* (coding for Death Receptor 4; DR4) [49], *TNFRSF10B* (coding for Death Receptor 5; DR5) [49], *TNFRSF21* (coding for Death Receptor 6; DR6) [50], and *FADD* [49] (Figure 5B). Inspection of all four treatment groups indicated that NFV alone accounted for a substantial component of the induction of several stress- and death-associated transcripts, particularly within the ER stress-associated module, with the combination treatment showing similar or further increased expression for several genes. Given that NFV alone has previously been linked to suppression of pro-survival signaling, particularly through the inhibition of the PI3K-Akt pathway [15], we next examined whether these transcriptomic data support downregulation of cell survival. KEGG gene set enrichment analysis demonstrated significant negative enrichment of the PI3K-Akt signaling pathway, and of the homologous recombination (HR) and Fanconi anemia DNA repair pathways in the CDDP/NFV combination-treated cells relative to vehicle controls (Figure 5C). However, HR- and Fanconi anemia-associated transcriptional programs are closely linked to cell-cycle progression, and their negative enrichment may partly reflect the suppression of proliferative programs observed in the same dataset rather than a functional reduction in DNA repair capacity [51,52]. Protein-level analysis further reinforced the transcriptomic findings. Immunoblotting confirmed that CDDP/NFV treatment increased the expression of the ER stress-induced protein CHOP and its downstream transcriptional target PUMA [53] relative to vehicle and single-agent controls in both PEO4 and IGROV-1/CP cells (Figure 5D). The combination treatment was also associated with reduced phospho-Akt (Ser473) relative to total Akt [54], with this reduction most evident in IGROV-1/CP cells. Because GSK-3β is a downstream target of Akt [54], we also examined its phosphorylation at Ser9 and observed reduced phospho-GSK-3β relative to the total GSK-3β in both cell lines (Figure 5E). Finally, NFV and combination treatment were associated with reduced abundance of the DNA repair protein 53BP1 [55], while phosphorylation of histone 2AX at Ser139 (γH2AX), a marker of double-strand DNA damage-associated signaling that can also arise during apoptotic DNA fragmentation [56], was increased with the drug combination compared to vehicle and single-agent controls (Figure 5F). Together, these data suggest that combined CDDP/NFV treatment is associated with ER stress, pro-apoptotic and death receptor-associated transcriptional changes, altered Akt signaling, and DNA damage-associated changes.

## 3. Discussion

**Figure 6:**
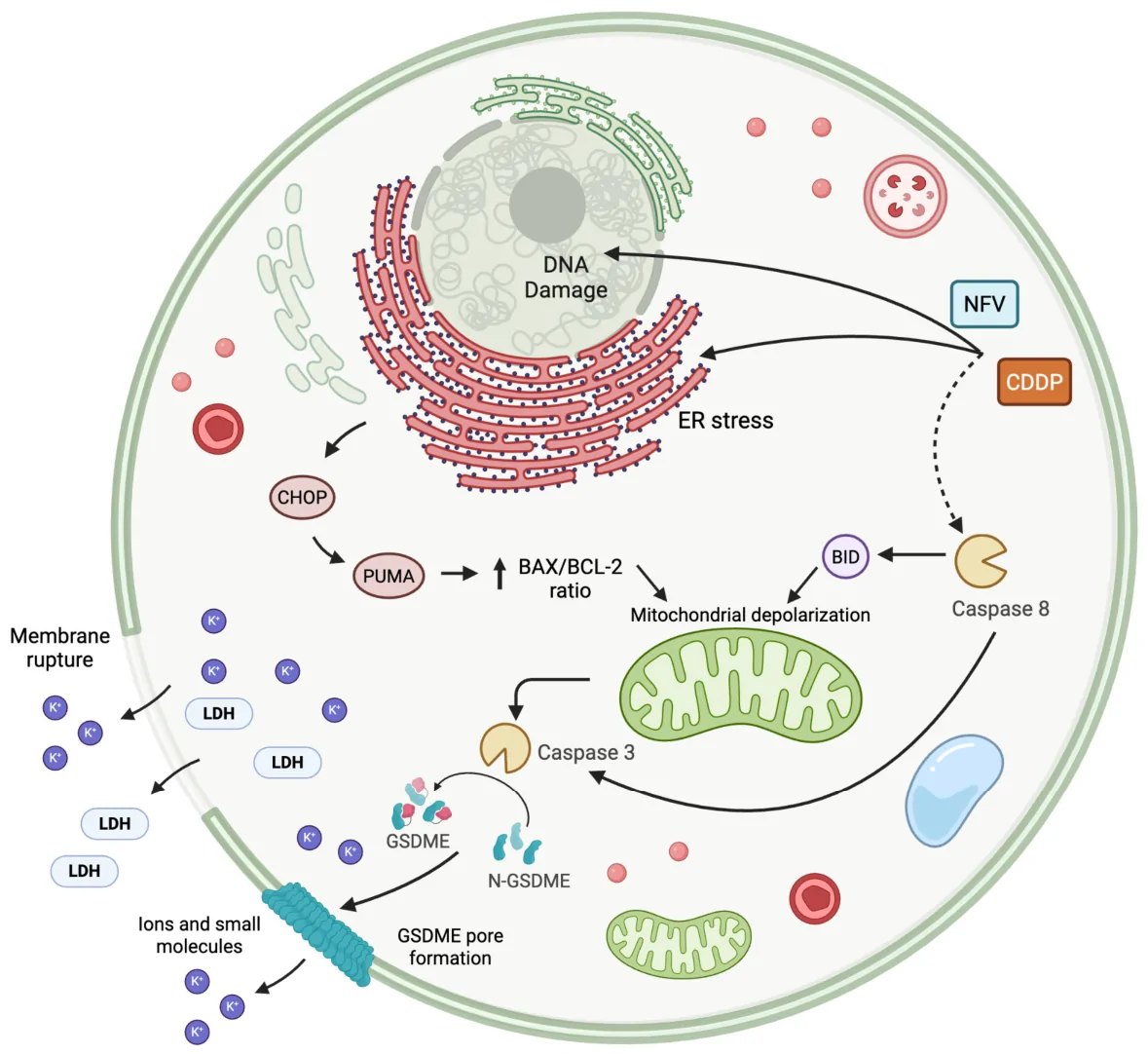
Proposed model of cellular responses associated with CDDP/NFV-induced lytic cell death in Pt-resistant ovarian cancer cells. The proposed relationships are a working hypothesis rather than an experimentally established sequence. Combined CDDP/NFV treatment is associated with DNA damage-related markers, endoplasmic reticulum (ER) stress, and activation of apoptotic signaling. ER stress is accompanied by increased CHOP and PUMA expression and a shift toward an increased BAX/BCL-2 ratio, while caspase-8 cleavage is associated with BID processing and mitochondrial apoptotic signaling. These changes coincide with mitochondrial depolarization and caspase-3 activation. Caspase-3 activity coincides with cleavage of gasdermin E (GSDME); the resulting N-terminal fragment (N-GSDME) can form membrane pores that release small molecules in established biological contexts, but their requirement for membrane permeabilization in this model was not determined. Combination treatment is also associated with terminal plasma membrane rupture and release of larger intracellular molecules such as lactate dehydrogenase (LDH). Created in BioRender. Forgie, B. (2026) https://BioRender.com/cbvagss (accessed on 20 September 2026).

In this study, NFV and CDDP demonstrated a synergistic cytotoxic interaction across three Pt-resistant ovarian cancer cell lines. Detailed characterization in PEO4 and IGROV-1/CP cells showed that the combination treatment produced a lytic cell death phenotype characterized by Annexin V/7-AAD positivity, caspase-3 and PARP cleavage, GSDME processing, LDH release, and plasma membrane-disruptive morphology. Pharmacological inhibition further supported contributions of caspase-8 and caspase-3 and was consistent with an association between their activity and GSDME processing. The response occurred in a cellular context marked by ER stress and pro-apoptotic transcriptional changes, altered Akt-associated markers, and DNA damage-associated changes. Together, these findings support a working model in which combined CDDP/NFV treatment engages caspase-associated signaling and is accompanied by GSDME processing and a lytic cell death phenotype, while the specific molecular requirements and temporal ordering of these events remain to be established (Figure 6).

These findings are relevant to the challenge of treatment resistance in ovarian cancer, where Pt-based chemotherapy remains a cornerstone of treatment but is frequently undermined by recurrence and the eventual emergence of Pt-resistant disease [11]. In many cases, Pt resistance reflects multiple adaptive mechanisms that can reduce treatment sensitivity and limit efficient execution of cell death programs [4,57]. In this context, drug repurposing strategies that intensify responses to Pt-derivatives with clinically available agents are especially attractive [58]. NFV has emerged as a promising repurposing candidate because, beyond its original antiviral role, it has been linked to ER stress induction, disruption of proteostatic homeostasis, altered pro-survival signaling, and enhanced anticancer activity in multiple model systems, including our work in high-grade serous ovarian cancer [14,15,59,60]. These data extend this work by showing that CDDP and NFV interact synergistically in the Pt-resistant ovarian cancer cells tested and by characterizing the associated lytic death phenotype.

Transcriptomic and protein-level analyses indicated that this cytotoxic response occurred in the context of pronounced cellular stress. CDDP/NFV-treated IGROV-1/CP cells showed enrichment of the UPR, apoptosis, ROS-associated signaling, TNF/NF-κB signaling, and p53-associated transcriptional programs, together with increased expression of stress- and apoptosis-associated proteins such as CHOP and PUMA. Examination of the individual treatment groups further suggested that NFV contributes significantly to several of these transcriptional responses, particularly the induction of ER stress-associated genes. This is consistent with NFV’s established ability to disrupt proteostatic homeostasis and induce the UPR [15,61], and suggests that NFV-associated cellular stress represents one component of the broader response observed during CDDP/NFV combination treatment. Together, these findings suggest that NFV and CDDP may contribute complementary effects to the combination response. NFV strongly engages ER stress-associated programs and is associated with altered Akt-related signaling, whereas CDDP provides a complementary DNA-damaging insult. The convergence of these distinct but potentially overlapping stress signals may reduce the capacity of Pt-resistant ovarian cancer cells to tolerate treatment-associated damage and thereby favor progression toward apoptotic and lytic cell death. Although these transcriptomic findings are supported by select protein-level changes, future studies validating selected ER stress- and death receptor-associated transcripts would help further characterize the molecular responses associated with the combined CDDP/NFV treatment.

A notable feature of the combination-associated death response was the simultaneous presence of apoptotic signaling and GSDME processing. Caspase-3 is a central executioner of apoptosis but can also cleave GSDME to generate a pore-forming N-terminal fragment, providing a molecular link between apoptotic execution and lytic cell death [62]. In cells expressing GSDME, this cleavage can promote membrane permeabilization, providing a mechanism through which apoptotic caspase activity can acquire lytic cell death features. This may limit the ability of damaged cells to recover from treatment-associated stress and thereby favor a more terminal cell death outcome [62,63,64]. Related work in lung adenocarcinoma has also linked NFV-based treatment to caspase-3 and GSDME cleavage, suggesting that engagement of this axis may extend beyond ovarian cancer, although the upstream context may differ across tumor types [65]. These findings identify GSDME processing as a reproducible molecular event associated with the lytic phenotype induced by the CDDP/NFV combination. However, GSDME loss-of-function studies will be required to determine if GSDME is necessary for membrane permeabilization and lysis.

Caspase-8 also emerged as an important component of the combination-induced response. CDDP/NFV treatment increased caspase-8 cleavage, while pharmacological inhibition of caspase-8 improved viability and reduced caspase-3 and GSDME cleavage. Although pharmacological inhibition at a late treatment endpoint does not definitively establish pathway ordering, these findings support involvement of caspase-8-associated signaling in the cell death response, without establishing whether caspase-8 activation precedes or follows other treatment-induced events. Caspase-8 is positioned at the interface of extrinsic and intrinsic apoptotic pathways and can amplify mitochondrial apoptosis through Bid processing while also contributing directly to executioner caspase activation [66]. The reduction in full-length Bid, the increase in the Bax/Bcl-2 ratio, and the mitochondrial depolarization we observed here are consistent with this type of apoptotic pathway amplification [38]. In parallel, RNA-seq analysis revealed increased expression of death receptor-associated transcripts, including DR4, -5, and -6, and FADD, raising the possibility that the treatment-associated transcriptional state is permissive for death receptor-linked or death receptor-like caspase-8 activation [67,68]. CHOP may be particularly relevant in this context, as unresolved ER stress can increase DR5 expression through CHOP activity and promote death-inducing signaling complex (DISC) formation, thereby linking the ER stress response to the extrinsic apoptotic signaling pathway [69,70]. However, the precise upstream trigger remains unresolved. ER stress, ROS accumulation, death receptor priming, and ligand-independent death receptor signaling may each contribute to the activation of caspase-8 and warrant further investigation [68,69,71,72]. Taken together, these observations support a possible contribution of caspase-8 to apoptotic and lytic signaling; however, the proposed sequence of events remains a working hypothesis requiring time-resolved and genetic validation.

Another interesting feature of the response of Pt-resistant ovarian cancer cells to the CDDP/NFV combination was the emergence of a p53-associated pro-apoptotic transcriptional program despite the mutant *TP53* background of PEO4 and IGROV-1/CP cells. *TP53* mutation and loss of normal p53 function are common features of ovarian cancer, particularly HGSOC [1,73,74]. Combination-treated cells showed enrichment of the p53 pathway and increased expression of transcripts including *BBC3*, *GADD45A/B*, and *AEN*, which are commonly associated with canonical p53-mediated stress responses [75]. These findings are unlikely to reflect restoration of wild-type p53 activity and instead more likely represent activation of alternative stress-responsive transcriptional circuitry that converges on components of classical p53 output. CHOP may contribute to this response, as ER stress-associated CHOP activity can promote apoptotic priming by inducing BH3-only proteins, including PUMA, BIM, and NOXA [43,53,69,76]. Consistent with this interpretation, increased PUMA expression was also observed at the protein level in both p53-mutant cell lines examined. Thus, CDDP/NFV treatment is associated with a p53-like pro-apoptotic transcriptional state even in the setting of impaired p53 function, highlighting convergence between cellular stress signaling and apoptotic effector programs.

CDDP/NFV treatment was also associated with changes in Akt- and DNA damage-related markers. Comparison of the phospho- and total-protein blots showed that NFV-containing treatments were associated with reduced phospho-Akt (Ser473) and phospho-GSK-3β (Ser9) relative to total Akt and GSK-3β respectively. These patterns are consistent with altered Akt-associated signaling. Akt activity has been linked to CDDP resistance by promoting cell survival, sustaining metabolic adaptation, and opposing apoptotic execution [10,77], but functional involvement of Akt in the present drug interaction was not directly tested. In parallel, CDDP/NFV treatment increased γH2AX levels, while NFV, alone and in combination with CDDP, reduced 53BP1 abundance. These observations indicate altered DNA damage-associated signaling but do not fully establish accumulation of unrepaired DNA breaks or impaired repair capacity [55,56]. In particular, γH2AX may also accumulate during apoptosis-associated DNA fragmentation [56]; its increase at the late treatment endpoint therefore cannot be attributed exclusively to primary DNA damage. The negative enrichment of HR and Fanconi anemia pathways should also be interpreted cautiously. Although these pathways are critical for repairing CDDP-induced DNA lesions [78,79], their transcriptional activity is also linked to cell-cycle progression [51,52], and reduced enrichment may partly reflect the strong suppression of proliferative programs following treatment. Restoration of HR capacity, including through BRCA reversion mutations that restore wild-type function such as those reported in PEO4 cells [80], is a recognized mechanism of acquired Pt resistance [81], but functional DNA repair assays would be required to determine whether the pathway changes observed here contribute directly to the CDDP/NFV cytotoxic interaction.

Several limitations warrant acknowledgment. First, these findings are based on in vitro Pt-resistant ovarian cancer models, and it remains to be determined whether the CDDP/NFV combination promotes similar death programs in vivo, particularly within the context of the tumor microenvironment. In vivo studies will also be necessary to evaluate the antitumor efficacy and tolerability of the drug combination under physiologically relevant conditions. The study also did not include matched Pt-sensitive counterparts, and therefore cannot determine whether the CDDP/NFV interaction is specific to the Pt-resistant state. Second, the caspase experiments rely on pharmacological inhibition at relatively high inhibitor concentrations and do not establish genetic requirement. Most protein and cell death measurements were also obtained at a late treatment endpoint, limiting conclusions regarding temporal ordering or whether changes reflect initiating events rather than downstream consequences. Furthermore, the requirement for GSDME for membrane disruption and cell lysis was not directly tested. Likewise, the RNA-seq analysis remains primarily descriptive. Although transcriptomic changes associated with ER stress and PI3K-Akt signaling were supported by protein-level changes in CHOP, PUMA, and phosphorylated Akt, respectively, further validation of additional targets, particularly those involved in death receptor signaling, would strengthen our understanding of the molecular mechanisms underlying the response to combined CDDP/NFV treatment. Third, although ferrostatin-1 and necrostatin-1 did not rescue viability under the conditions tested, pathway-specific target-engagement controls were not included and ferroptotic or necroptotic contributions therefore cannot be definitively excluded. This caution may be particularly relevant because NFV alone can induce ER stress-associated ferroptotic cell death in other cellular contexts [82], suggesting that the dominant death program engaged by NFV may vary by cellular background and by whether NFV is used as a single agent or in combination with chemotherapy [82]. Finally, clinical pharmacokinetic studies have reported substantial interindividual variability in NFV exposure, with peak total plasma concentrations ranging from approximately 4–26 µM in patients receiving standard and escalated doses [61,83,84]. However, NFV is extensively (> 98%) protein-bound in circulation [85], and the sustained 20 µM exposure used here may not reflect the unbound drug concentrations achieved clinically. The 20 µM concentration was selected as a standardized in vitro treatment condition with limited single-agent cytotoxicity in the models studied. Further investigation of clinically relevant exposure durations, tumor penetration, and the effect of protein binding will be necessary to assess the translational potential of this combination. The present study also examined CDDP rather than carboplatin, the more commonly used Pt agent in ovarian cancer [86]. While both drugs have similar mechanisms of action in cancer cells, carboplatin is less reactive than CDDP and is generally better tolerated systemically, which has contributed to its widespread clinical use [87]. These considerations highlight the importance of evaluating the CDDP/NFV interaction across clinically relevant exposure profiles and determining whether similar activity is observed with carboplatin. Within these limitations, our findings demonstrate a reproducible synergistic cytotoxic interaction between NFV and CDDP in Pt-resistant ovarian cancer cell models and identify an associated lytic death phenotype involving caspase-8/caspase-3 signaling and GSDME processing. These data provide a foundation for further genetic, time-resolved, and pharmacological evaluation of the combination and support additional preclinical investigation of NFV as a repurposed combination partner to enhance CDDP cytotoxicity in Pt-resistant ovarian cancer.

## 4. Materials and Methods

### 4.1. Cell Culture and Reagents

This study used the PEO4, IGROV-1/CP, and TOV21G cell lines as models of Pt-resistant ovarian cancer. PEO4 cells were isolated from the ascites of a patient 10 months after their second round of Pt-based chemotherapy and are considered resistant to clinically achievable concentrations of Pt [31]. IGROV-1/CP cells were derived from the IGROV-1 parental line and were rendered Pt-resistant through chronic exposure to increasing concentrations of CDDP [32]. TOV21G cells were isolated from a patient before Pt exposure [88] and have been shown to be intrinsically Pt-resistant [89]. The PEO4 and TOV21G cell lines were kindly gifted by Dr. Taniguchi (Fred Hutchinson Cancer Center, University of Washington, Seattle, WA, USA) and Dr. Mes-Masson (Centre de recherche du Centre Hospitalier de l’Université de Montréal (CHUM), Montréal, QC, Canada), respectively. IGROV-1/CP was obtained from Dr. Stephen Howell (University of California, San Diego, CA, USA). PEO4 cells harbor a homozygous inactivating *TP53* mutation [90], whereas IGROV-1/CP cells carry heterozygous inactivating *TP53* mutations inherited from the parental line [90,91], and TOV21G cells are wild-type for *TP53* [90]. All cells were cultured in RPMI 1640 media (Mediatech, Manassas, VA, USA) supplemented with 10% fetal bovine serum (FBS) (Corning Inc, Corning, NY, USA), 0.01 mg/mL of human insulin (Roche, Indianapolis, IN, USA), 10 mM HEPES (Corning), 100 IU penicillin (Mediatech), 100 µg/mL streptomycin (Mediatech), 2 mM L-Alanyl-L-glutamine (Glutagro^TM^, Corning), and 1 mM sodium pyruvate (Corning). Cells were maintained at 37 °C in a humidified incubator with 5% CO_2_. To complete this study, the following drugs were used: nelfinavir mesylate hydrate (NFV) (Sigma Chemical Co., St. Louis, MO, USA), cis-diamminedichloroplatinum II (CDDP) (Sigma), Z-IETD-FMK (Cedarlane Laboratories, Burlington, ON, Canada), Z-DEVD-FMK (Cedarlane), necrostatin-1 (NEC-1) (Cedarlane), and ferrostatin-1 (FER-1) (Cedarlane). CDDP was dissolved in 0.9% sodium chloride (saline) (Sigma). NFV, Z-IETD-FMK, Z-DEVD-FMK, NEC-1, and FER-1 were dissolved in dimethyl sulfoxide (DMSO) (Sigma), with a maximum concentration of 0.1% (*v*/*v*) used in cell culture.

### 4.2. Assessment of Cell Proliferation and Viability

Cell proliferation and viability were assessed through microcapillary cytometry using the Guava Muse Cell Analyzer (Cytek Biosciences, Fremont, CA, USA). Cells were seeded in 6-well plates and treated with increasing concentrations of CDDP for 3 h (h). Thereafter, the CDDP-containing media were removed, and the cells were incubated for an additional 72 h in the presence of either a constant concentration of 20 µM NFV or drug-free media. The cells were then trypsinized, collected, and stained with the Muse Count and Viability Reagent (Cytek) according to the manufacturer’s protocol. In brief, cells were stained simultaneously with a membrane-permeable dye to obtain the total cell count, and a membrane-impermeable dye to determine the number of cells with decreased membrane integrity (non-viable cells). From these counts, the number of viable cells with intact plasma membranes was determined. Dose–response curves were generated from viability measurements, and CDDP IC50 values were calculated for each cell line in the absence or presence of 20 µM NFV using nonlinear regression in GraphPad Prism version 11.0.0 (Dotmatics, Boston, MA, USA). For pharmacological inhibition experiments, PEO4 and IGROV-1/CP cells were treated with CDDP (10 µM) for 3 h followed by NFV (20 µM) for 72 h in the absence or presence of the caspase-3 inhibitor Z-DEVD-fmk (100 µM), the caspase-8 inhibitor Z-IETD-fmk (100 µM), the ferroptosis inhibitor ferrostatin-1 (FER-1; 1 µM), or the RIPK1-dependent necroptosis inhibitor necrostatin-1 (NEC-1; 10 µM). Cell viability was assessed as described above.

### 4.3. Cell Vitality Measurement and Drug–Drug Interaction Assessment

Drug interaction between CDDP and NFV was assessed using the Loewe additivity model in SynergyFinder version 3.20.0 [30,92], with complementary analyses using the Bliss independence and zero interaction potency (ZIP) reference models. In this model, scores less than −10 indicate antagonism between two drugs, whereas scores from −10 to 10 suggest an additive interaction, and scores greater than 10 reflect a synergistic drug interaction [92]. Cells were seeded in 96-well plates and treated with a concentration-combination matrix of CDDP and NFV. CDDP was administered for 3 h and then removed, after which cells were exposed to NFV for 72 h. We tested CDDP concentrations of 2.5, 5, and 10 µM in combination with NFV concentrations of 2.5, 5, 10, and 20 µM, evaluating all combinations. Cell vitality [93] was measured using the Cell Counting Kit-8 (CCK-8, Dojindo, Kumamoto, Japan) assay according to the manufacturer’s instructions, and absorbance values were recorded at 450 nm. Vitality values were normalized to vehicle-treated controls, which were set to 100% vitality. These normalized vitality data were then entered into SynergyFinder to analyze the drug interaction.

### 4.4. Annexin-V and 7-AAD Staining

Phosphatidylserine exposure on the outer leaflet of the plasma membrane was assessed using the Muse Annexin V & Dead Cell Kit (Cytek) on the Muse Cell Analyzer according to the manufacturer’s protocol. In brief, following treatment, the floating and adherent cells were collected, centrifuged, resuspended in fresh media, and stained with Annexin V & Dead Cell Reagent for 20 min (min) at room temperature (RT). Cell populations were quantified as live, early apoptotic, late apoptotic, and dead cells.

### 4.5. Brightfield Microscopy

Cellular morphology was assessed by brightfield microscopy following treatment with vehicle, CDDP, NFV, or CDDP/NFV under the treatment conditions described above. Representative images were acquired at the experimental endpoint using a 10× objective to assess treatment-associated morphological changes, including cell detachment, swelling, vacuolization, and ballooning.

### 4.6. Mitochondrial Membrane Depolarization

We evaluated depolarization across the inner mitochondrial membrane (IMM) following drug treatment using the Muse MitoPotential Kit (Cytek) and the Muse Cell Analyzer following the manufacturer’s instructions. Following treatment and harvesting of floating and adherent cells, the samples were centrifuged, resuspended in 1X assay buffer, and stained with the Muse MitoPotential working solution for 20 min at 37 °C followed by incubation with the 7-AAD dye for 5 min at RT. Cells were classified by IMM polarization status and viability, and the percentage of dead/dying cells with a depolarized IMM was quantified.

### 4.7. LDH Release Assay

Cell death-associated plasma membrane lysis was assessed by measuring LDH release into culture supernatants using the Cytotoxicity Detection Kit (LDH) (Roche, Mannheim, Germany), according to the manufacturer’s instructions. Briefly, cells were seeded in 96-well plates, and following treatment, supernatants were collected and incubated with the LDH reaction mixture for 30 min at RT in the dark. Absorbance values were measured at 490 nm. To assess the contribution of terminal plasma membrane disruption to LDH release, PEO4 and IGROV-1/CP cells were treated with CDDP/NFV in the absence or presence of glycine (5 mM) [37], and LDH release was measured as described above.

### 4.8. Reactive Oxygen Species Measurement

Intracellular reactive oxygen species (ROS) production was assessed using the Muse Oxidative Stress Kit (Cytek) and the Muse Cell Analyzer according to the manufacturer’s instructions. PEO4 and IGROV-1/CP cells were exposed to vehicle, CDDP, NFV, or CDDP/NFV treatment regimens as previously described and analysed at the 72 h end point. Following treatment, floating and adherent cells were collected, centrifuged, resuspended, and stained with the Muse Oxidative Stress Reagent. Samples were then analyzed using the Muse Cell Analyzer, and the percentage of ROS-positive cells was quantified.

### 4.9. Immunoblotting

After treatment incubation, ovarian cancer cells were collected by scraping into ice-cold 1X PBS and pelleted by centrifugation. Cell pellets were stored at −80 °C until protein extraction. We lysed cells in NP-40 lysis buffer supplemented with protease inhibitors. Lysis was done on ice for 30 min, after which lysates were clarified by centrifugation at 12,000× *g* for 15 min at 4 °C, and protein concentrations were determined using the Pierce BCA protein assay (Thermo Fisher Scientific, Waltham, MA, USA). Twenty-five µg of total protein per lane was resolved at 200 V for 35 min on 12% SDS-polyacrylamide gels prepared using the TGX Stain-Free FastCast Acrylamide Kit (Bio-Rad Laboratories, Hercules, CA, USA). Proteins were transferred onto PVDF membranes using the Trans-Blot Turbo Transfer System for 7 min (Bio-Rad). Membranes were blocked in 5% non-fat milk in TBS-T for 1 h at RT and incubated overnight at 4 °C with primary antibodies diluted 1:1000 in 5% non-fat milk in TBS-T or 5% Bovine Serum Albumin (BSA, Sigma) in TBS-T. The following primary antibodies were used for immunoblotting analysis: caspase-3 (#9622, Cell Signaling Technology, Danvers, MA, USA), PARP (#9542, Cell Signaling), gasdermin E (#84005, Cell Signaling), caspase-8 (#9746, Cell Signaling), Bid (#2002, Cell Signaling), Bcl-2 (#3498, Cell Signaling), Bax (#5023, Cell Signaling), CHOP (#2895, Cell Signaling), PUMA (#12450, Cell Signaling), phospho-AKT (Ser473) (#9271, Cell Signaling), Akt (#2920S, Cell Signaling), phospho-GSK-3β (Ser9) (#5558, Cell Signaling), GSK-3β (#12456, Cell Signaling), phospho-histone H2AX (Ser139) (γH2AX) (#9718, Cell Signaling), 53BP1 (#612523, BD Transduction Laboratories, Franklin Lakes, NJ, USA), and β-actin (A5441, Sigma). After washing in TBS-T, membranes were incubated with the appropriate HRP-conjugated secondary antibodies, goat anti-rabbit IgG (H + L) HRP conjugate (1706515, Bio-Rad) or goat anti-mouse IgG (H + L) HRP conjugate (1706516, Bio-Rad), for 1 h at RT. Protein bands were visualized using Clarity Western ECL Substrate (Bio-Rad) and imaged with a ChemiDoc Touch Imaging System version 1.2.0.(Bio-Rad). Cleaved GSDME bands were quantified by densitometry using Bio-Rad Image Lab software version 6.1. Background-adjusted band intensities were normalized to the corresponding β-actin loading controls.

### 4.10. RNA Sequencing and Analysis

IGROV-1/CP cells were treated in three independent biological replicates per condition with vehicle, 10 µM CDDP for 3 h followed by culture in drug-free media for 24 h, 20 µM NFV for 24 h, or 10 µM CDDP for 3 h followed by culture in 20 µM NFV-containing media for 24 h. We then submitted cell pellets to Genome Québec (Montreal, QC, Canada) for RNA extraction, library preparation, and sequencing. Total RNA was extracted using the RNeasy Plus Universal Mini Kit (QIAGEN, Hilden, Germany, cat. no. 73404) according to the manufacturer’s instructions. Briefly, the lysis buffer reagent QIAzol was added to the cell samples, and homogenization was performed by passing the lysate 15 times through a 20-gauge needle attached to a sterile 3 mL syringe. RNA was eluted in 35 µL of the buffer provided with the extraction kit. Total RNA was quantified and its integrity assessed using the 5K/RNA/Charge Variant Assay LabChip and RNA Assay Reagent Kit (PerkinElmer, Shelton, CT, USA). Libraries were generated from 250 ng of total RNA using the Illumina Stranded mRNA Prep Kit (Illumina, San Diego, CA, USA), including mRNA enrichment, according to the manufacturer’s recommendations. Libraries were quantified using the KAPA Library Quantification Kit (Kapa Biosystems, Roche), and average fragment size was determined using a Fragment Analyzer 5300 (Agilent Technologies, Santa Clara, CA, USA). Libraries were normalized, pooled, denatured in 0.02 N NaOH, neutralized with pre-load buffer, and loaded at 170 pM onto an Illumina NovaSeq X Plus 25B lane for 2 × 100 bp paired-end sequencing. A PhiX control library was spiked in at 1%. Base calling and demultiplexing were performed using BCL Convert v4.2.4 to generate FASTQ files. Gene-level count tables generated using the DRAGEN pipeline were used for downstream analysis. Differential expression analysis was performed in R version 4.5.3using DESeq2 (v1.46.0) [94]. Principal component analysis and sample-to-sample correlation analysis were used to assess replicate clustering and consistency. Genes with an adjusted *p*-value < 0.05 and |log2 fold change| ≥ 1 were considered significantly differentially expressed. Gene set enrichment analysis was performed in R using fgsea (v1.32.4) [95] with genes ranked by the Wald statistic from DESeq2 differential expression analysis. Hallmark and Gene Ontology Biological Process gene sets were obtained from the Molecular Signatures Database (MSigDB v2025.1.Hs) [96] using msigdbr (v25.1.1). KEGG [97] pathway gene sets were retrieved using KEGGREST (v1.46.0) and analyzed using the same fgsea-based preranked enrichment workflow.

### 4.11. Statistical Analysis

Data are presented as mean ± SD from three independent experiments unless otherwise indicated. We performed statistical analyses using GraphPad Prism (Dotmatics). Statistical significance among groups was determined using one-way ANOVA followed by Tukey’s post hoc multiple comparisons test. Where appropriate, two-way ANOVA followed by Sidak’s multiple comparisons test was used. A *p*-value, or a false discovery rate (FDR)-adjusted *p*-value (adj *p*), <0.05 was considered statistically significant.

## Data Availability

The data presented in this study are openly available in the NCBI Gene Expression Omnibus (GEO) repository, accession number GSE338229.

## Supplementary Materials

The following supporting information can be downloaded at https://www.mdpi.com/article/10.3390/ph19101665/s1: Figure S1: Additional synergy analysis of combined cisplatin and nelfinavir treatment in platinum-resistant ovarian cancer cells: Synergy heatmaps generated using the (**A**) Bliss independence and (B) zero interaction potency (ZIP) reference models in PEO4, IGROV-1/CP, and TOV21G cells. Cells were treated with cisplatin (CDDP; 2.5, 5, or 10 µM) for 3 h, followed by nelfinavir (NFV; 2.5, 5, 10, or 20 µM) for 72 h. Heatmaps were generated using SynergyFinder software from normalized cell vitality data from CCK8 assays. Synergy scores greater than 10 indicate synergistic interactions, scores between −10 and 10 indicate additivity, and scores less than −10 indicate antagonism; Figure S2: Densitometric quantification of GSDME cleavage following combined cisplatin and nelfinavir treatment and caspase-3 inhibition. (**A**) Quantification of cleaved gasdermin E (GSDME) in PEO4 and IGROV-1/CP cells following treatment with vehicle, cisplatin (CDDP; 10 µM, 3 h), nelfinavir (NFV; 20 µM, 72 h), or CDDP followed by NFV. (**B**) Quantification of cleaved GSDME in PEO4 and IGROV-1/CP cells following CDDP/NFV treatment in the absence or presence of the caspase-3 inhibitor Z-DEVD-fmk (100 µM). Band intensities were quantified using Bio-Rad Image Lab software and normalized to the corresponding β-actin loading controls. Data are presented as mean ± SD from 2 independent biological experiments; Figure S3: RNA-seq quality control demonstrates appropriate sample clustering and replicate consistency. (**A**) Principal component analysis (PCA) of variance-stabilized transcriptomic data from IGROV-1/CP cells treated with vehicle, 10 µM cisplatin (CDDP) for 3 h, 20 µM nelfinavir (NFV) for 24 h, or 10 µM CDDP for 3 h followed by 20 µM NFV for 24 h. Samples clustered according to treatment condition, with principal component 1 (PC1) and principal component 2 (PC2) accounting for 67% and 12% of the total variance, respectively. (**B**) Sample-to-sample correlation heatmap generated from variance-stabilized transcriptomic data showing strong within-group correlation and overall consistency among biological replicates across treatment conditions; Figure S4: CDDP/NFV treatment increases accumulation of reactive oxygen species in ovarian cancer cells. (**A**,**B**) Reactive oxygen species (ROS) levels were measured in PEO4 (**A**) and IGROV-1/CP (B) cells following treatment with vehicle, 10 µM cisplatin (CDDP) for 3 h, 20 µM nelfinavir (NFV) for 72 h, or the combination, using the Muse Oxidative Stress Assay. In PEO4 cells, the CDDP/NFV combination increased the percentage of ROS-positive cells relative to vehicle and monotherapy controls. In IGROV-1/CP cells, ROS levels were also elevated following combination treatment, although the response was comparable to that observed with CDDP alone. Data are presented as mean ± SD from 3 independent experiments. Statistical analysis was performed using one-way ANOVA with Tukey’s multiple comparisons test. *** *p* < 0.001 versus vehicle; ### *p* < 0.001 versus CDDP + NFV; ns, not significant.
