# Supplementary material for: Nelfinavir Synergizes with Cisplatin and Promotes GSDME-Associated Lytic Cell Death in Platinum-Resistant Ovarian Cancer Cells
Source: Pharmaceuticals (Basel). 2026 Oct 21;19(10):1665. doi: 10.3390/ph19101665 (PMC13708070; doi:10.3390/ph19101665)
Supplement: Supplementary file 1 [file pharmaceuticals-19-01665-s001.zip › pharmaceuticals-4618788-supplementary.pdf]

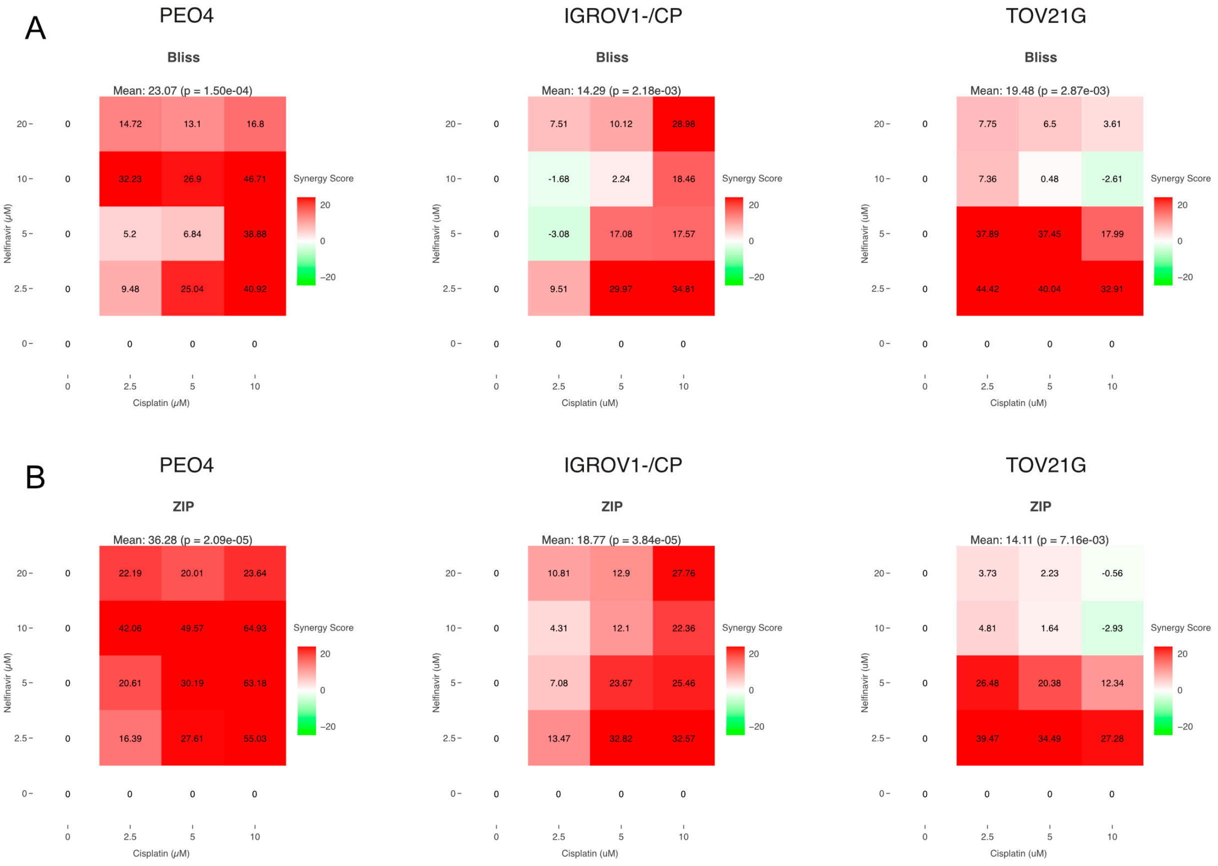

Supplementary Figure S1. Additional synergy analysis of combined cisplatin and nelfinavir treatment in platinum-resistant ovarian cancer cells. Synergy heatmaps generated using the (A) Bliss independence and (B) zero interaction potency (ZIP) reference models in PEO4, IGROV-1/CP, and TOV21G cells. Cells were treated with cisplatin (CDDP; 2.5, 5, or 10 μM) for 3 h, followed by nelfinavir (NFV; 2.5, 5, 10, or 20 μM) for 72 h. Heatmaps were generated using SynergyFinder software from normalized cell vitality data from CCK8 assays. Synergy scores greater than 10 indicate synergistic interactions, scores between −10 and 10 indicate additivity, and scores less than −10 indicate antagonism.

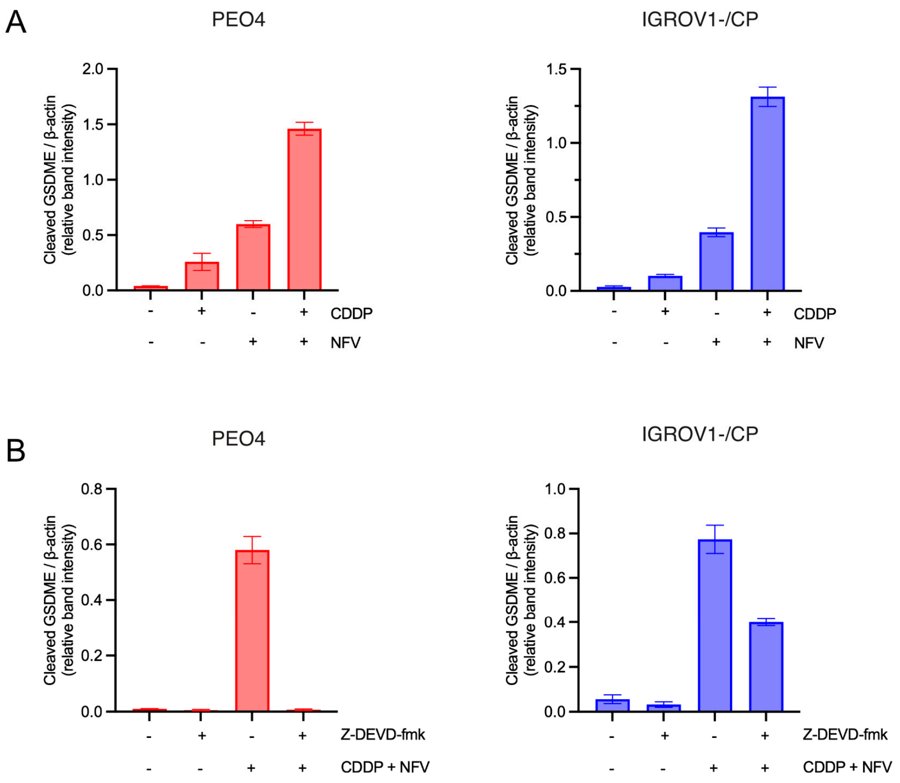

Supplementary Figure S2. Densitometric quantification of GSDME cleavage following combined cisplatin and nelfinavir treatment and caspase-3 inhibition. (A) Quantification of cleaved gasdermin E (GSDME) in PEO4 and IGROV-1/CP cells following treatment with vehicle, cisplatin (CDDP; 10 µM, 3 h), nelfinavir (NFV; 20 µM, 72 h), or CDDP followed by NFV. (B) Quantification of cleaved GSDME in PEO4 and IGROV-1/CP cells following CDDP/NFV treatment in the absence or presence of the caspase-3 inhibitor Z-DEVD-fmk (100 µM). Band intensities were quantified using Bio-Rad Image Lab software and normalized to the corresponding β-actin loading controls. Data are presented as mean ± SD from 2 independent biological experiments.

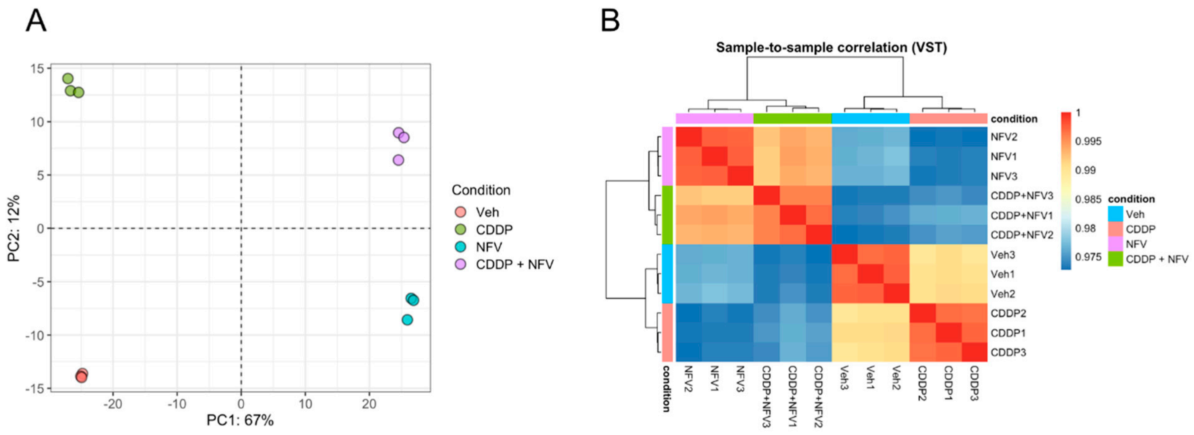

Supplementary Figure S3. RNA-seq quality control demonstrates appropriate sample clustering and replicate consistency. (A) Principal component analysis (PCA) of variance-stabilized transcriptomic data from IGROV-1/CP cells treated with vehicle, 10 µM cisplatin (CDDP) for 3 h, 20 µM nelfinavir (NFV) for 24 h, or 10 µM CDDP for 3 h followed by 20 µM NFV for 24 h. Samples clustered according to treatment condition, with principal component 1 (PC1) and principal component 2 (PC2) accounting for 67% and 12% of the total variance, respectively. (B) Sample-to-sample correlation heatmap generated from variance-stabilized transcriptomic data showing strong within-group correlation and overall consistency among biological replicates across treatment conditions.

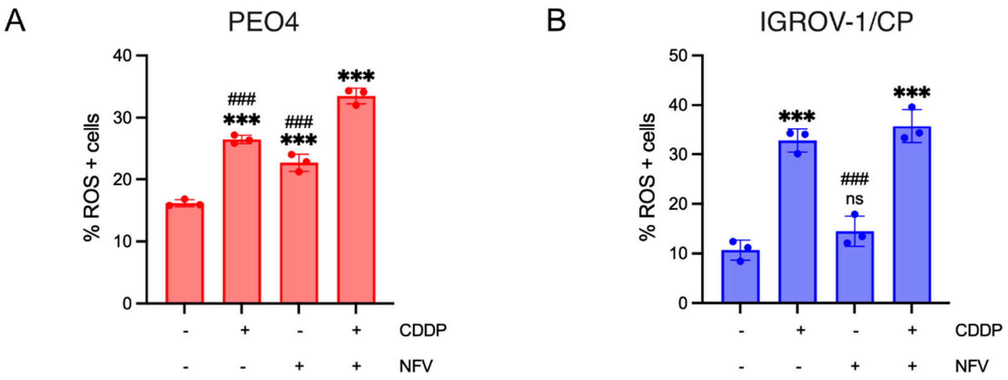

Supplementary Figure S4. CDDP/NFV treatment increases accumulation of reactive oxygen species in ovarian cancer cells. (A, B) Reactive oxygen species (ROS) levels were measured in PEO4 (A) and IGROV-1/CP (B) cells following treatment with vehicle, 10 µM cisplatin (CDDP) for 3 h, 20 µM nelfinavir (NFV) for 72 h, or the combination, using the Muse Oxidative Stress Assay. In PEO4 cells, the NFV/CDDP combination increased the percentage of ROS-positive cells relative to vehicle and monotherapy controls. In IGROV-1/CP cells, ROS levels were also elevated following combination treatment, although the response was comparable to that observed with CDDP alone. Data are presented as mean ± SD from 3 independent experiments. Statistical analysis was performed using one-way ANOVA with Tukey's multiple comparisons test. *** $p < 0.001$ versus vehicle; ### $p < 0.001$ versus CDDP + NFV; ns, not significant.
